# Vitamin D Status and Its Correlation With Anthropometric and Biochemical Indicators of Cardiometabolic Risk in Serbian Underground Coal Miners in 2016

**DOI:** 10.3389/fnut.2021.689214

**Published:** 2021-08-19

**Authors:** Ivana Šarac, Jovica Jovanović, Manja Zec, Zoran Pavlović, Jasmina Debeljak-Martačić, Milica Zeković, Jelena Milešević, Mirjana Gurinović, Maria Glibetić

**Affiliations:** ^1^Laboratory for Public Health Nutrition, Centre of Research Excellence in Nutrition and Metabolism, Institute for Medical Research, National Institute of Republic of Serbia, University of Belgrade, Belgrade, Serbia; ^2^Faculty of Medicine, Department of Occupational Health, University of Niš, Niš, Serbia; ^3^Department of Sanitary Chemistry and Ecotoxicology, Institute for Public Health Požarevac, Požarevac, Serbia

**Keywords:** coal miners, Vitamin D, physical activity, cardiometabolic health, metabolic syndrome, obesity, lipids

## Abstract

The status of vitamin D in underground working coal miners and its association with their cardiometabolic health is rarely studied. This study aimed to examine vitamin D (VitD) status in Serbian underground coal miners and to correlate it with anthropometric and laboratory indicators of cardiometabolic risk. Nutritional data (food frequency questionnaire, FFQ, and two times repeated 24 h recall), anthropometric data (including segmental analysis by bio-impedance analyzer TANITA BC-545N), arterial tension, and biochemical and hematological data of 103 coal miners (aged 22–63 years) were correlated with their late summer (early September) serum 25 (OH)D levels (measured by HPLC). 68.9% of the studied coal miners were overweight/obese, and 48.5% had metabolic syndrome. Their mean VitD nutritional intakes were low: 5.3 ± 3.8 μg/day (FFQ) and 4.9 ± 8 μg/day (24 h recalls), but their mean serum 25 (OH)D levels were surprisingly high (143.7 ± 41.4 nmol/L). Only 2.9% of the coal miners had 25(OH)D levels lower than 75 nmol/L (indicating an insufficient/deficient status), while 63.2% had values above 125 nmol/L (upper optimal limit), and even 10.7% had values above 200 nmol/L. There were no statistical differences in 25 (OH)D levels in the coal miners with or without metabolic syndrome (or overweight/obesity). Interestingly, 25(OH)D levels had significant positive correlations with body mass index (BMI), fat mass (FM), fat mass percentage (FM%), limbs FM%, serum triglycerides, GGT, AST, ALT, and ALT/AST ratio, and had significant negative correlations with serum HDL-cholesterol and age. All these correlations were lost after corrections for age, FM, FM%, and legs FM%. In Serbian coal miners, high levels of early September VitD levels were observed, indicating sufficient non-working-hour sun exposure during the summer period. Furthermore, the unexpected positive correlations of VitD levels with anthropometric and biochemical parameters indicative of obesity, metabolic syndrome, and fatty liver disease were found. More research is needed on the VitD status of coal miners (particularly in the winter period) and its relationship with their cardiometabolic status.

## Introduction

The relationship between vitamin D (VitD) status and obesity, metabolic syndrome, diabetes, and cardiovascular diseases has gained scientific attention over the last decades. Low serum vitamin D levels have been associated with obesity, metabolic syndrome, and increased risk for diabetes and cardiovascular diseases (1–4). However, inconsistent findings exist, and cause/effect relationships still need to be confirmed (1–5).

On the other hand, in parallel with the increasing worldwide prevalence of obesity, metabolic syndrome, diabetes, and cardiovascular diseases (6–8), with the latter two being among the first four leading causes of death across the world, there is an increasing prevalence of VitD insufficiency/deficiency (9, 10). Some populations are at increased risk for low VitD levels (10–12). The modern lifestyle in big cities, with high buildings, air pollution, and low insolation of streets; everyday commuting by closed vehicles and predominantly indoor work and leisure activities (without enough outdoor activities and direct exposure to the sun), leads to an increased prevalence of VitD insufficiency/deficiency among populations (10, 13) where it was not expected to appear (e.g., in regions with low latitude and high insolation) (14, 15). Some professions and working conditions are more associated with low VitD levels. For example, shift work, night work, and working indoors, without direct sun exposure, are associated with lower VitD serum levels (16, 17). All of those are characteristics of underground mining work. Nevertheless, insufficient data exist on the serum levels of VitD in this population of workers (17–21), and the data do not show the lower levels compared with the general population or surface workers from the same geographic region (17–21). Therefore, more data are needed on this topic. Furthermore, there are insufficient data on the cardiometabolic status of coal miners, with only few recently published studies (22–26).

Given that, this study aimed to examine the following: (1) the levels of serum VitD among a sample of underground working coal miners in Serbia; (2) the anthropometric and biochemical indicators of cardiometabolic risk among them; (3) the association of VitD levels with indicators of cardiometabolic risk.

## Materials and Methods

### Participants and Study Design

This cross-sectional study included 103 male coal miners, aged 22–63 years, from the underground Brown Coal Mine “Rembas” (shaft “Vodna,” Strmosten), in the Resavica coal mining basin in Serbia, who during the period May 9, 2016, to September 9, 2016, (late summer) underwent their periodic (annual) preventive systematic medical examinations, obliged by the law and performed by the Institute of Occupational Medicine Niš. The study was a joint project of the Institute of Medical Research of the University of Belgrade and the Institute of Occupational Medicine Nis and supported by the Ministry of Education, Science and Technological Development of the Republic of Serbia. The study was in accordance with the ethical principles of the Declaration of Helsinki and approved by the Institute of Occupational Health Niš Ethics Board.

According to official data (27), in 2016, in Serbia, there were 2,750 underground coal miners, working in shafts, and the vast majority of them were employed in the Resavica basin (76.4%). The required study sample size was 45 subjects, which was calculated according to the previously published data on summer Serbian general male population VitD levels (28) and the decided precision (margin of error) of ± 5 nmol/L at 95% confidence level, and the formula: needed sample size = (1.96)^2^^*^ (SD)^2^/(decided precision)^2^ (29). Considering possible unfitness, dropout, and planned further correlation analyses, we decided to initially include (i.e., invite to participate in the study) all 140 employed miners in this coal mine who attended their preventive systematic medical examinations, which would represent about 5% of Serbian coal miners.

On the day of the examination, the coal miners attended the center for preventive medical checkups at 07:00, after fasting overnight for 12 h. Detailed examinations to obtain information on their health were performed by medical specialists (including specialists of occupational health and internal medicine), and incorporated, *inter alia*, blood pressure measurements and fasting blood, and urine sampling for laboratory biochemical analyses. Supplementary analyses for this study involved the following: (1) anthropometric measures (including, among others segmental body composition analysis by bioelectrical impedance, BIA); (2) dietary surveys [such as the two times repeated 24 h recall and FFQ, which was validated for VitD intake (30)], (3) and further analyses of the obtained biochemical serum samples to determine VitD status by high-performance liquid chromatography (HPLC).

From the 140 coal miners who attended the medical preventive checkups and were invited to participate in the study, a total of 103 agreed to participate in the study, signed written consent, and performed all the tests and analyses required for this study (i.e., the participants who did not manage to perform all of the required examinations, presented here, or fulfilled the exclusion criteria, were excluded from the study). The exclusion criteria were the following: the presence of serious medical conditions; established diagnosis of diabetes mellitus with regular use of glucose-lowering therapy; established diagnosis of hyperlipidemia with regular use of lipid lower drugs; a known liver disease with non-metabolic etiology, excluding non-alcoholic fatty liver disease (NAFLD); taking any antihypertensive and glucose/lipid-lowering agents in the previous 24 h).

All the underground miners worked in three rotating shifts (2 days in first shift: 07:00–15:00; 2 days in second shift: 15:00–23:00; 2 days in third shift: 23:00–07:00; then, 2 days off) and always stayed underground, in the shaft during their shifts. They all performed heavy physical work (31), such as hewing coal, loading coal, and erecting roof supports.

### Anthropometric Measurements and Measurement of Arterial Tension

Measurements of body height, body weight, fat mass (FM), fat mass percentage (FM%), fat-free mass (FFM), muscle mass, bone mass, waist circumference, and hip circumference were taken by a trained medical doctor, following standard techniques (32, 33). The subjects were measured fasted at 8 am, after emptying their bladder and bowels, wearing only underwear. Body height was measured to the nearest 0.01 m with a wall-mounted stadiometer. Bodyweight, FM, FM%, FFM, muscle mass, and bone mass were measured on a portable scale Tanita Inner Scan V BC-545N Segmental Body Composition Monitor (Tanita, Aerolit d.o.o., Belgrade, Serbia), by BIA. The body weight was measured to the nearest 0.1 kg. The measurements were performed according to the recommendations provided by the manufacturer. Additional segmental analyses included muscle mass of trunk, arms, and legs; fat percentage of the trunk, arms, and legs; and visceral fat level (VFL), calculated according to the Tanita algorithm by the BIA segmental body analysis (34, 35). Body mass index (BMI: kg/m^2^) was calculated by dividing the weight (in kilograms) by the square of height (in meters), and according to their BMI, the coal miners were classified into three groups: normal weight (BMI: 18.5–24.9 kg/m^2^), overweight (BMI: 25–29.9 kg/m^2^), and obese (BMI 30 kg/m^2^) (36). Increased adiposity was defined according to %FM ≥ 21–22 (depending on age) (37). Waist and hip circumferences were measured with a flexible, stretch-resistant tape, to the nearest centimeter. Waist circumference was measured at the approximate midpoint between the lower margin of the last palpable rib and the top of the iliac crest, at minimum respiration. Hip circumference was measured at the widest points over the hip at the level of the greater trochanter, with the tape parallel to the floor (33). Abdominal obesity was defined by waist circumference ≥ 102 cm (33). Supplementary calculations included waist to hip ratio (WHR), and WHR ≥0.91 was considered as indicative of centripetal obesity, i.e., truncal fat distribution (33).

Arterial blood pressures were measured in the morning, after sitting for at least 10 min, on the left upper arm, with a calibrated mechanic sphygmomanometer and stethoscope (Becton Dickinson, Franklin Lakes, NJ, United States), and the average of two measurements was presented as the result. Hypertension was defined by systolic blood pressure ≥ 140 mmHg and/or diastolic blood pressure ≥ 90 mmHg, while pre-hypertension was defined by systolic blood pressure between 130 and 140 mmHg and/or diastolic blood pressure between 85 and 90 mmHg (38).

### Laboratory Biochemical Analyses of Blood Samples

Fasting blood samples were collected between 07:00 and 08:00 in specific Vacutest Kima® blood collection tubes, available from Vacutest Kima® S.R.L., Italy (Yunycom d.o.o., Belgrade, Serbia), including the following: Vacutest® Kimased sodium citrate 3.8% 1.6 ml tubes (for erythrocyte sedimentation rate, tubes, erythrocyte sedimentation rate (ESR), analysis), Vacutest® K3EDTA 3 ml tubes (for blood cell count, hematocrit, and hemoglobin analysis), Vacutest® Serum Separator Clot Activator 6 ml tubes for analysis of serum glucose, total cholesterol, high-density lipoproteins (HDL)-cholesterol and low-density lipoproteins (LDL)-cholesterol, triglycerides, urea, creatinine, total bilirubin, aspartate aminotransferase (AST), alanine aminotransferase (ALT), alkaline phosphatase (ALP), gamma-glutamyl transferase (GGT), and VitD.

Following the instructions of the manufacturer for processing and handling of the blood collection tubes, ESR analyses were performed with the Westergren's method at 18°C, using the Kimased ESR rack with scaling. One-hour Westergren values were determined by reading the distance (in mm) from the plasma meniscus to the top of the column of sedimented erythrocytes 30 min, immediately (maximum 1 h) after blood collection. Analyses of blood cell counts, hematocrit, and hemoglobin were performed on Phoenix NCCNCC-2310 (NeoMedica d.o.o., Nis, Serbia), and the serum coagulation tubes (after allowing them to clot for at least 30 min in an upright position at room temperature) were centrifuged at 3,000 × g for 10 min at room temperature. Following centrifugation, standard photometric assays were performed using an ARCHITECT c8000 Abbott clinical chemistry analyzer (Abbott Laboratories S.A., Belgrade, Serbia), and commercially available kits (Abbott Laboratories S.A., Belgrade, Serbia). Intra- and inter-assay coefficients of variation (CVs) for all measurements were <5%.

The criteria for the existence of diabetes and impaired fasting glucose were set according to the American Diabetes Association (ADA) 2016 guidelines (39). The existence of dyslipidemia was established based on the National Cholesterol Education Program—Adult Treatment Panel III (NCEP-ATP III) criteria from 2001 (40). The atherogenic risk was also calculated as the ratio of total cholesterol/HDL-cholesterol, the ratio of LDL-cholesterol/HDL-cholesterol, and the ratio of triglycerides/HDL-cholesterol, as all of these ratios have clinical implications (41, 42). Metabolic syndrome was defined according to the American Heart Association/National Heart, Lung, and Blood Institute (AHA/NHLBI) 2005 criteria (43).

### Serum VitD Analysis by HPLC Method

Serum total 25 (OH) vitamin D, calculated as a sum of 25 (OH) vitamin D2 and 25 (OH) vitamin D3, was determined using an Agilent 1100 series HPLC system equipped with a diode array detector (DAD) and the RECIPE's (RECIPE Chemicals + Instruments GmbH, Munich, Germany) ClinRep® HPLC Complete Kit for 25 (OH) VitD2/D3 in plasma and serum (containing a ClinTest® Test Solution, ClinCal® Serum Calibrators, ClinChek® Serum Controls, sample preparation vials, the precipitation agent, internal standard, mobile phase, and analytical column), according to the instructions of the manufacturer.

Immediately after the analyses with the ARCHITECT c8000 clinical chemistry analyzer were performed, 1.5 ml of serum was separated from the serum coagulation tubes in 2 ml Eppendorf microtubes and kept frozen at −80°C in a freezer until further VitD assays were performed.

After 24 months of storage at −80°C (which should not influence the results, since vitamin D is quite stable during prolonged storage at −80°C, up to 6–30 years) (44, 45), serum samples were defrosted and vortex-mixed and 0.4 ml of the samples were transferred to the sample preparation vials, filled with 0.5 ml of the precipitation agent. Afterward, 0.4 ml of internal standard was added (cooled at temperature −20°C), and the vials were vortex-mixed for 30 s and centrifuged for 5 min at 10,000 × g. After centrifugation, 50 μl of the upper liquid phase of the supernatant was injected into the Aligent 1100 HPLC system, with a flow rate of 1 ml/min, and the column heater at 40°C. The sample components were separated on the analytical column, and the analytes, 25 (OH) VitD2 and 25 (OH) VitD3, were detected with the DAD detector at a wavelength of 264 nm, with retention times of 7.3 and 6.4 min, respectively. Quantification was performed comparing the peak areas of 25 (OH) VitD2 and 25 (OH) VitD3 with the peak area of the internal standard (its retention time was 8.3 min). In addition, the ClinChek® Serum Controls were used after running every 10 examined samples to perform quality control. The precision of measurement (expressed as CVs) for 25 (OH) VitD2 and 25 (OH) VitD3 was 6.5 and 6%, respectively.

Taking into consideration different cutoff values for defining VitD deficiency/insufficiency (9, 10, 46–48), the serum levels of 25 (OH) VitD below 50 nmol/L were defined as a deficient state, while those between 50 and 75 nmol/L were defined as an insufficient state (9). Deficiency below 50 nmol/L was defined as mild to moderate and below 30 nmol/L was defined as severe (10). The values above 175 nmol/L were defined as mildly increased, above 200 nmol/L was defined as moderately increased (the lowest literature-reported level associated with toxicity in subjects with normal renal function and without primary hyperparathyroidism), and above 250 nmol/L was defined as excessive (hypervitaminosis) (46). The values between 75 and 175 nmol/L were defined as VitD sufficiency (“normal range”), while the values between 75 and 125 nmol/L were defined as “optimal.” (46, 49).

### Assessment of Dietary Intakes

Dietary intake data were collected by a trained medical doctor using a validated food frequency questionnaire (FFQ) (30) and the two times repeated 24 h recall method, in accordance with the European Food Safety Authority (EFSA) EU Menu methodology (32, 50). The FFQ and the first 24 h recall were performed on the day of physical examination and blood sampling, while the second 24 h recall was performed with a minimum of 7 days apart, by a telephone call. The average intakes of energy, macronutrients, and VitD were calculated with the DIET ASSESS & PLAN (DAP) software (51, 52), which was evaluated by the EFSA (32, 50) using the national validated Food Atlas for Portion Size Estimation (53) and Serbian Food Composition Database, developed in compliance with EuroFIR standards (54). Calculated data for VitD intakes were evaluated by comparison with the adequate intake (AI) defined by the EFSA in 2016 (i.e., minimum 15 μg/day for adults) (48).

### Statistical Analysis

Statistical analyses were performed using the SPSS 22.0 (SPSS Inc., Chicago, IL, United States) statistical software. Data are presented as the mean ± SD, along with minimum and maximum values. The normality of data distribution was tested by the Kolmogorov–Smirnov test. Differences in VitD levels between subjects with and without overweight/obesity or metabolic syndrome were analyzed by the Mann–Whitney *U*-test. Correlation between VitD levels and biochemical data, anthropometric measurements, daily energy, and macronutrient/micronutrient nutrient intake was assessed by Pearson's correlation coefficient (for parametric data) and Spearman's correlation coefficient (for non-parametric data). Partial correlations were used when one or more controlling variables were included in the analyses. Linear stepwise regression models were made to explore the strength and independence of associations between VitD levels and characteristics of analyzed subjects. *P*-value was expressed as a number rounded up to three decimal places; statistical significance was assumed at a two-tailed *P* < 0.05.

## Results

### Descriptive Statistics: Characteristics of the Studied Population of Coal Miners

Table 1 presents general, anthropometric, and blood pressure data, Table 2 presents the data on biochemical analyses, and Table 3 gives the data on dietary intakes of energy, macronutrients, and VitD.

**Table 1:** Demographic, anthropometric, and arterial BP data of the studied coal miners (*N* = 103, men), and correlation with serum 25 (OH) vitamin D levels.

	**Descriptives**			**Correlation with vitamin D levels** **(non-adjusted)**	**Correlation with vitamin D levels** **(adjusted for age, FM, and FM%)**	**Correlation with vitamin D levels** **(adjusted for age, FM, FM%, and legs FM%)**
	***Mean***	***SD***	***(Min–Max)***	***r***	***r***	***r***
Age (years)	40.6	9.6	(22.0–63.0)	**−0.283** ^**^		
Body height (cm)^§^	177.4	6.5	(162.0–196.0)	0.014	−0.116	−0.124
Body weight (kg)	86.0	16.6	(49.4–126.3)	**0.201** ^*^	0.068	0.034
BMI (kg/m^2^)	27.2	4.5	(16.5–38.1)	**0.243** ^*^	**0.205** ^*^	0.185
FM (kg)	18.0	8.9	(2.7–39.2)	**0.210** ^*^		
FM% (%)	19.9	7.2	(5.0–36.2)	**0.200** ^*^		
FFM (kg)	68.1	9.8	(40.6–88.8)	0.150	0.068	0.034
Muscle mass (kg)	64.7	9.4	(38.4–84.6)	0.151	0.070	0.036
Skeletal mass (kg)	3.4	0.5	2.1–4.3)	0.159	0.081	0.048
**Segmental analysis**						
Arms FM% (%)	18.1	5.9	(4.6–39.4)	0.162	−0.076	−0.137
Trunk FM% (%)	21.2	8.6	(5.0–35.9)	0.171	−0.163	0.148
Legs FM% (%)	18.4	6.4	(3.8–42.7)	**0.250** ^*^	**0.208** ^*^	
Arms muscle mass (kg)	7.8	1.4	(3.7–12.1)	**0.194** ^*^	0.149	0.090
Trunk muscle mass (kg)	35.2	5.1	(21.6–48.8)	0.120	0.078	0.022
Legs muscle mass (kg)	21.7	3.2	(13.1–28.9)	0.182	0.007	0.046
VFL	8.0	4.2	(1.0–18.5)	0.077	0.046	0.167
Waist circumference (cm)	98.9	12.9	(69.0–129.0)	0.143	−0.032	−0.050
Hip circumference (cm)	104.4	8.3	(86.0–124.0)	**0.211** ^*^	0.091	0.074
WHR	0.95	0.07	(0.78–1.11)	0.021	−0.098	−0.104
Systolic BP (mmHg)^§^	127.1	14.2	(100.0–160.0)	0.059	0.091	0.105
Diastolic BP (mmHg)^§^	83.1	8.0	(60.0–120.0)	−0.005	0.034	0.025

§ *Spearman's rank correlation coefficients—r_s_ (otherwise—data represent Pearson's correlation coefficients—r)*.

*Bolded coefficients are statistically significant*.

* *p < 0.05; correlation is significant at the 0.05 level (two-tailed)*.

** *p < 0.01; correlation is significant at the 0.01 level (two-tailed)*.

*BMI, body mass index; FM, fat mass; FM%, percentage of fat mass; FFM, fat-free mass; VFL, visceral fat level; WHR, waist to hip ratio; BP, blood pressure; SD, standard deviation; r, Pearson's correlation coefficients; r_s_, Spearman's rank correlation coefficients; p, statistical significance of correlations*.

**Table 2:** Hematological and biochemical data of the studied coal miners (*N* =103, men), and correlation with serum 25 (OH) vitamin D levels.

	**Descriptives**			**Correlation with vitamin D levels** **(non–adjusted)**	**Correlation with vitamin D levels** **(adjusted for age, FM, and FM%)**	**Correlation with vitamin D levels** **(adjusted for age, FM, FM%, and legs FM%)**
	***Mean***	***SD***	***(Min–Max)***	***r***_***s***_	***r***_***s***_	***r***_***s***_
ESR (mm/h)	6.8	3.5	(3.0–22.0)	−0.112	−0.037	−0.026
WBC (× 10^9^/L)	7.8	2.1	(3.8–14.4)	0.038	0.018	0.003
RBC (× 10^12^/L)	5.0	0.5	(3.2–6.3)	**0.208** ^*^	0.129	0.096
HGB (g/L) ^§^	155.1	10.1	(135.0–183.0)	0.142	0.073	0.047
HCT (L/L)^§^	0.4	0.0	(0.3–0.5)	0.144	0.112	0.096
MCV (fL)^§^	87.8	5.1	(69.3–102.4)	−0.169	−0.036	−0.013
MCH (pg)	30.9	2.4	(23.0–41.2)	−0.108	−0.059	−0.058
MCHC (g/L)	352.7	17.7	(324.0–430.0)	0.022	−0.065	−0.077
PLT (× 10^9^/L)	240.2	56.7	(131.0–427.0)	−0.028	0.001	−0.012
Glucose (mmol/L)	6.0	1.2	(3.5–11.5)	−0.015	−0.030	−0.037
Triglycerides (mmol/L)	2.0	1.0	(0.5–4.9)	**0.249** ^*^	**0.237** ^*^	0.190
Total cholesterol (mmol/L)	5.8	1.3	(3.5–9.8)	0.169	**0.198** ^*^	0.135
LDL-cholesterol (mmol/L)	3.4	1.1	(1.1–7.5)	0.118	0.134	0.071
HDL-cholesterol (mmol/L)	1.3	0.4	(0.7–2.7)	**-0.250** ^*^	**−0.209** ^*^	−0.145
TC/HDL	5.0	2.0	(1.5–11.5)	**0.300** ^**^	**0.293** ^**^	**0.220** ^*^
LDL/HDL	2.9	1.4	(0.4–7.4)	**0.245** *	**0.235** *	0.159
TG/HDL	1.9	1.4	(0.2–7.1)	**0.273** ^**^	**0.251** ^**^	0.192
AST (U/L)	24.5	7.5	(12.0–64.0)	**0.235** *	0.194	0.191
ALT (U/L)	30.3	14.8	(11.0–93.0)	**0.351** ^**^	**0.254** ^*^	0.187
ALT/AST	1.23	0.43	(0.58–2.44)	**0.276** ^*^	0.170	0.089
GGT (U/L)	37.3	31.0	(10.0–245.0)	**0.203** ^*^	0.165	0.107
ALP (U/L)	56.3	16.4	(14.0–99.0)	0.084	−0.002	0.007
Total bilirubin (μmol/L)	11.7	6.6	(3.7–35.4)	−0.169	−0.191	−0.198
Urea (mmol/L)	4.9	1.0	(2.8–8.3)	0.036	0.097	0.089
Creatinine (μmol/L)	72.8	10.1	(54.5–120.3)	**0.244** ^*^	**0.207** ^*^	0.157

§ *Pearson's correlation coefficients—r (otherwise—data represent Spearman's rank correlation coefficients—r_s_)*.

*Bolded coefficients are statistically significant*.

* *p < 0.05; correlation is significant at the 0.05 level (two-tailed)*.

** *p < 0.01; correlation is significant at the 0.01 level (two-tailed)*.

*FM, fat mass; FM%, percentage of fat mass; ESR, erythrocyte sedimentation rate; WBC, white blood cell count; RBC, red blood cell count; HGB, hemoglobin; HCT, hematocrit; MCV, mean volume; MCH, mean corpuscular hemoglobin; MCHC, mean corpuscular hemoglobin concentration; PLT, platelet count; LDL, low-density lipoprotein; HDL, high -density lipoprotein; TC/HDL, total cholesterol/HDL-cholesterol ratio; LDL/HDL, LDL-cholesterol/HDL-cholesterol ratio; TG/HDL, triglycerides/HDL-cholesterol ratio; AST, aspartate aminotransferase; ALT, alanine aminotransferase; ALP, alkaline phosphatase; GGT, gamma glutamyl transferase; SD, standard deviation; r_s_, Spearman's rank correlation coefficients; r, Pearson's correlation coefficients; p, statistical significance of correlations*.

**Table 3:** Nutritional intakes data of the studied coal miners (*N* =103, men), and correlation with serum 25 (OH) vitamin D levels.

	**Descriptives**			**Correlation with vitamin D levels** **(non-adjusted)**	**Correlation with vitamin D levels** **(adjusted for age, FM, and FM%)**	**Correlation with vitamin D levels** **(adjusted for age, FM, FM%, and legs FM%)**
	***Mean***	***SD***	***(Min–Max)***	***r***_***s***_	***r***_***s***_	***r***_***s***_
Total caloric intake (kcal/d)	3955.2	1095.4	(1609.0–7170.2)	0.119	0.117	0.078
Protein intake (kcal/d)	509.8	171.2	(186.2–1199.0)	0.056	0.050	0.022
Fat intake (kcal/d)	1851.6	565.6	(864.0–3547.5)	0.018	0.045	0.019
Carbohydrates intake (kcal/d)	1497.4	504.4	(492.4–2963.6)	0.167	0.144	0.106
Alcohol intake (kcal/d)	96.4	157.6	(0.0–886.9)	0.158	0.123	0.088
Total caloric intake (kcal/d)–day 1^§^	3957.8	1190.3	(2025.0–7284.4)	0.182	0.181	0.158
Protein intake (kcal/d)–day 1^§^	510.4	181.3	(228.8–1036.3)	0.116	0.121	0.101
Fat intake (kcal/d)—day 1^§^	1830.1	639.7	(514.8–3634.4)	0.080	0.081	0.072
Carbohydrates intake (kcal/d) – day 1^§^	1533.1	568.0	(419.0–3288.0)	**0.211** ^*^	0.186	0.162
Alcohol intake (kcal/d) – day 1^§^	84.3	188.0	(0.0–1108.8)	**0.225** ^*^	0.192	0.135
Vitamin D intake (μg/d)	4.9	8.0	(1.1–65.2)	−0.089	−0.063	−0.079
Vitamin D intake (μg/d) – day 1^§^	5.5	11.4	(0.9–98.0)	−0.040	−0.015	−0.033
Vitamin D intake FFQ (μg/d)^¥^	5.3	3.8	(1.2–18.5)	−0.007	−0.139	−0.170

§ *Data referring only to the first 24 h recall, representing the day before sampling of blood (otherwise—data represent the mean of two recalls)*.

¥ *Data derive from FFQ (otherwise—data derive from 24-h recalls, representing the mean of two recalls, or only referring to day 1, the day before sampling of blood)*.

*Bolded coefficients are statistically significant*.

* *p < 0.05; correlation is significant at the 0.05 level (two-tailed)*.

*FM, fat mass; FM%, percentage of fat mass; FFQ, food frequency questionnaire; SD, standard deviation; r_s_, Spearman's rank correlation coefficients; p, statistical significance of correlations*.

The mean age was 40.6 ± 9.6 years, but 10-year age categories were not represented equally, and only 11.7% of the participants were above 52-year-old (Supplementary Figure 1). More than half (53.5%) of the participants were smokers, and the huge majority (79.6%) lived in rural areas (the rest lived in semi-urban areas).

The average BMI belonged to the category of overweight (27.2 ± 4.5 kg/m^2^), and almost half of the participants (45.6%) were overweight, while about one-quarter (23.3%) were obese, with these two BMI categories making together above two-third of the studied population (68.9%). Less than one-third (28.2%) had normal weight, and only 2.9% were underweight. Almost half of the participants (45.6%) had increased adiposity, according to % FM ≥ 21–22 (depending on age). According to waist circumference, almost half of the participants (45.6%) had abdominal obesity, even though the average waist circumference value was under the cutoff value for abdominal obesity (i.e., < 102 cm). Centripetal obesity/fat distribution according to WHR was present in 75.7% of the participants.

The average measured values of fasting serum glucose (Table 2) were above the ADA cutoff values for the diagnosis of impaired fasting glucose (“pre-diabetes”), and 63.1% of the participants had serum glucose values above this value (i.e., ≥5.6 mmol/L), while at the same time 11.7% of the participants also met the criteria for diabetes (i.e., fasting serum glucose ≥ 7 mmol/L). Also, the average blood lipid levels were above the ATP III cutoff values for triglycerides (i.e., ≥1.69 mmol/L), total cholesterol (i.e., ≥5.17 mmol/L), and LDL-cholesterol (i.e., ≥3.34 mmol/L), while the average HDL-cholesterol levels were below the recommended values for men (i.e., <1.03 mmol/L) (Table 2). In total, 53.4, 62.1, and 45.6% of the participants had elevated triglycerides, total cholesterol, and LDL-cholesterol, respectively, while only 26.2% of the participants had reduced HDL-cholesterol levels. In total, 84 participants (81.6%) had dyslipidemia according to at least one of the ATP III criteria, while 11 participants (10.7%) fulfilled all the ATP III criteria for dyslipidemia (at the same time having increased triglycerides and LDL-cholesterol levels, and decreased HDL-cholesterol levels). The ratios of total cholesterol, LDL-cholesterol, and triglycerides to HDL-cholesterol were increased (i.e., respectively, ≥5, ≥3.3, and ≥0.87) in 43.7, 34, and 73.8% of the participants, respectively.

The average systolic and diastolic pressures were in the normal range, but 51.5% of the participants met the criteria for pre-hypertension or hypertension.

Only three participants used lipid-lowering agents (but they did not use them regularly), while 11 participants used antihypertensive drugs. No other medicaments used were reported, and no medications were used on the morning of the examination.

According to the obtained data on glucose, triglycerides, HDL-cholesterol, blood pressure, and waist circumference (or regular use of relevant medicaments), 48.5% of the participants fulfilled the 2005 AHA criteria for the diagnosis of metabolic syndrome. The serum levels of ALT above 30 U/L, GGT above 30 U/L, and ALT/AST ratio higher than 1.25 [indicative for NAFLD (55–57)] had 36.9, 48.5, and 40.8% of the participants, respectively.

The average energy intake, according to the repeated 24 h recalls, was 3955.2 ± 1095.4 kcal/day, which is in accordance with existing recommendations (58), but there was a huge inter-individual difference (individual intakes ranged from 1,600 to 7,200 kcal) (Table 3). On average, proteins, fats, carbohydrates, and alcohol provided 12.9 ± 2.1, 46.8 ± 6.7, 38.1 ± 7.1, and 2.2 ± 3.5% of total daily energy intake, respectively. Daily alcohol intake (average for 2 days, according to 24 h recalls) varied from 0 g (in 54.4% of coal miners) to 126.7 g. Similarly, according to FFQs, only 58.3% of the coal miners reported alcohol intake.

The average intake of VitD was quite low and far below the recommended values by EFSA: 5.3 ± 3.8 μg/day (according to FFQs) and 4.9 ± 8 μg/day (according to 24 h recalls) (Table 3). Only two of the participants had an adequate nutritional intake of VitD, above 15 μg/day. There was a statistically significant correlation between VitD intakes by FFQs and 24 h recalls (Spearman's rho correlation coefficient 0.268, *p* = 0.04). Only four of the coal miners consumed supplements of VitD.

### Vitamin D Status

The average value of serum 25(OH) vitamin D was way above the lower cutoff value for the sufficient state: 143.7 ± 41.4 nmol/L, ranging from 43.5 to even 247.5 nmol/L. Only three (2.9%) of the coal miners had levels of VitD lower than 75 nmol/L (indicating an insufficient/deficient status, with two subjects having mild deficiency), while 63.2% had values above 125 nmol/L (upper optimal limit). In total, 22.3% of the coal miners had 25(OH) VitD above 175 nmol/L (the upper limit for normal range), and even 10.7% had values above 200 nmol/L (the lowest literature-reported level associated with toxicity). None of the coal miners had VitD values above 250 nmol/l (hypervitaminosis) (For graphical presentation, refer to Supplementary Figure 2.)

Most of the total serum 25 (OH) VitD was 25(OH) vitamin D3 (143.5 ± 41.4 nmol/L, ranging from 43.5 to 247.5 nmol/L), while 25(OH) VitD2 made only a small part (0.2 ± 1.6 nmol/L, ranging from 0 to 15 nmol/L).

The results on 25 (OH) VitD levels were not much different if the subjects who consumed supplements of VitD were excluded from the analysis (mean total serum 25 (OH) VitD was 144.1 ± 42.1 nmol/L, again ranging from 43.5 to 247.5 nmol/L). Considering the four coal miners who consumed the supplements, their VitD levels ranged from 105.5 to 149.5 nmol/L (in average 123.2 ± 18.7 nmol/L), only nine subjects were sunbathing by the seaside during the summer, and the levels of VitD were statistically marginally higher in them: 172.3 ± 46.5 vs. 141 ± 40.1 nmol/L (Mann–Whitney test *p* = 0.05).

There was no statistically significant difference in VitD levels between those who lived in rural and semi-urban areas (141.6 ± 42.2 vs. 152.1 ± 37.6 nmol/L, respectively), or smokers and non-smokers 140.8 ± 43.2 vs. 146.4 ± 40.3 nmol/L, respectively).

There was no correlation between serum 25 (OH) VitD levels and VitD nutritional intake, according to both FFQs and 24 h recalls (Table 3).

When only nutritional data from the day before blood sampling were analyzed, VitD levels correlated with 24 h recall carbohydrates and alcohol intake the previous day (Table 3).

### Association of VitD Levels With Cardiometabolic (Anthropometric and Biochemical) Parameters

There were no statistically significant difference in 25 (OH) VitD levels in the coal miners with or without metabolic syndrome, or with or without overweight/obesity (148.9 ± 42.0 vs. 138.8 ± 40.5 nmol/L, and 147 ± 37.1 vs. 136.4 ± 49.5 nmol/L, respectively, Mann–Whitney test *p* > 0.05), even though in subjects with those conditions VitD levels seemed somehow higher.

Tables 1, 2 show the correlation of VitD levels with cardiometabolic (anthropometric and biochemical) parameters.

Serum 25 (OH) vitamin D levels had significant negative correlations with age and HDL-cholesterol levels and had significant positive correlations with body weight, BMI, FM, FM%, limbs FM%, arms muscle mass, hip circumference, serum triglycerides, GGT, AST, ALT, total cholesterol/HDL-cholesterol, LDL-cholesterol/HDL-cholesterol, triglycerides/HDL-cholesterol ratios, and ALT/AST ratio (Tables 1, 2). Leg FM% was the anthropometric parameter that correlated most with VitD levels. At the same time, the above-mentioned biochemical variables had significant positive inter-correlations with anthropometric parameters indicative of increased adiposity (FM and FM%) and, surprisingly, some of them more with indicators of peripheral (gluteo-femoral) rather than with indicators of centripetal (truncal) adiposity (e.g., lipid levels correlated more with hip circumference and leg FM%, than with waist circumference and trunk FM%, Supplementary Table 1). Serum 25 (OH) VitD levels also positively correlated with erythrocytes count and creatinine (Table 3), while creatinine also had a positive correlation with muscular mass (Supplementary Table 1).

To eliminate the possible confounding, we performed Pearson's and Spearman's rank partial correlations. The correlations with lipids, creatinine, and ALT remained significant even after correcting for age, FM, and FM%, but were lost after additional corrections for leg FM% (with exception of total cholesterol/HDL-cholesterol ratio, which only remained significant correlation with VitD levels after this additional correction) (Table 3).

The stepwise linear regression analysis (which included age, and biochemical and anthropometric data as predictor variables), identified that the most significant predictors of VitD levels were age, leg FM%, and triglyceride levels (the models 1 and 2 have accounted for a statistically significant amount of VitD variance, and explained 23.8 and 23% of VitD variance, respectively) (Table 4).

**Table 4:** Linear (stepwise) regression models for assessing the impact of specific characteristics of coal miners (general, anthropometric, biochemical, and nutritional) on serum 25 (OH) vitamin D levels.

**Model**	***R^**2**^***	**Adjusted *R^**2**^***	**Coefficients**	**β**	***B***	**95% *CI* for *B***
						**Lower bound** **Upper bound**
1	0.268	0.238	(Constant)		58.609	[43.829, 73.388]^**^
			Age	−0.364	−0.630	[−0.947, −0.312]^**^
			Legs FM%	0.279	0.719	[0.216, 1.221]^**^
			Triglycerides	0.204	3.263	[0.433, 6.093]^*^
			ALT	0.136	0.152	[−0.056, 0.359]
2	0.252	0.230	(Constant)		62.273	[48.292, 76.254]^**^
			Age	−0.390	−0.674	[−0.987, −0.361]^**^
			Legs FM%	0.325	0.839	[0.361, 1.316]^**^
			Triglycerides	0.220	3.529	[0.707, 6.351]^*^

* *p < 0.05; regression coefficients are significant at the.05 level (two–tailed)*.

** *p < 0.01; regression coefficients are significant at the 0.01 level (two–tailed)*.

*FM%, percentage of fat mass; ALT, alanine aminotransferase; R^2^, coefficient of determination; β (beta), standardized linear regression coefficients; B, unstandardized linear regression coefficients; CI, confidence intervals; p, statistical significance*.

## Discussion

This study is among the very rare ones that examined the levels of VitD in a population of underground coal miners. We found surprisingly high levels of late-summer serum 25 (OH) VitD, which were much higher compared with the same season VitD data on an urban-living, general male Serbian population from similar geographic latitude (28), and previously published values for coal miners from other geographic regions (18–21).

Apart from this, to the best of our knowledge, this is the first published study that examined the association of VitD levels in coal miners with their cardiometabolic status. Again, we have found unexpected positive correlations with anthropometric data and biochemical parameters indicative of obesity, metabolic syndrome, and fatty liver disease, which is in contrast with previously published data (28, 59).

Such unexpected findings require explanation and more exploration.

The high levels of serum 25 (OH) vitamin D can be explained by the late summer season (beginning of September) when the serum levels of VitD are expected to be highest, and by living in rural/semi-urban areas, with high isolation, where additional agricultural work is quite common in that period (leading to additional sun exposure during non-coal mining working hours). Therefore, it would be necessary to examine the winter/early spring levels of VitD in the same group, when there would be much less sun exposure. The nutritional intake of VitD in this study was quite low, and far below the current EFSA recommendations, and only four subjects consumed VitD supplements. Therefore, it could be expected to find much lower values during winter/early spring.

However, the higher vitamin D levels can be also a consequence of their heavy physical work, leading to increased lipolysis and release of VitD from fat and muscular depots (60–62). Studies indicate that serum VitD levels increase after intensive physical activity (60, 63–65) and remain increased even after 24 h (60, 63, 64). People who exercise regularly, even indoors, have higher VitD levels compared with sedentary people (60, 65–67). The data on increased VitD levels after weight loss (accompanied by increased lipolysis) also support this hypothesis (68). This can additionally explain the difference in VitD status with the urban-living Serbian general male population, not performing hard physical work, in which levels were almost three times lower (49.8 ± 17.2 nmol/L), despite quite similar BMI (28 ± 6.3 kg/m^2^), waist circumference (98.5 ± 18 cm), and slightly higher FM% (24.8 ± 10.3%) (28). However, this cannot explain the difference in VitD status with other published data on summer VitD status in coal miners around the world (18–21). The main explanations for the difference with the Newcastle (UK) coal miners [where average values of 73.8 ± 73.4 nmol/L were found during the summer period (18), which were two times lower compared with these data] are geographic latitude and the lower level of insolation, apart from the earlier time of sampling (from May to July). In the study from India, samples were taken over a whole year, not specified if summer or winter (21), and in two other studies from UK and Turkey, the samples were from the winter/early spring period (19, 20). Nevertheless, the values in all those studies were much lower, particularly in Turkish workers (20).

However, there can be some additional explanations for the high vitamin D levels in the coal miners of this study.

In this study, vitD levels had a significant positive association with alcohol intake the previous day. Nevertheless, it is quite possible that the coal miners consciously withheld their alcohol consumption both in the 24 h recalls and FFQs (since it was an official medical checkup), and that their alcohol intake was much higher, since some data place this profession among the professions with highest alcohol consumption (21, 69, 70). Several cross-sectional studies showed positive associations of alcohol consumption with serum 25 (OH) VitD levels (66, 71–74). Even though these could be confounding findings, there can be a direct association between alcohol intake and VitD metabolism. It was shown in rats that chronic alcohol intake increases serum 25 (OH) VitD3, while it decreases serum 1, 25 (OH)_2_ VitD3 (75). The effect is most probably conveyed by a direct inhibition of parathyroid hormone (PTH) secretion by alcohol (76, 77). PTH stimulates the 1-alpha hydroxylation of 25 (OH) VitD3 to 1,25 (OH)_2_ VitD3. Nevertheless, only a small fraction of 25 (OH) VitD3 is converted to 1,25 (OH)_2_ VitD3 (78), and it is less likely that the inhibited 1-alpha hydroxylation significantly contributed to the considerably increased 25 (OH) VitD3 levels in this study. In accordance, in one study, only about a 10 nmol/L increase in serum 25 (OH) VitD was noted 5 years after parathyroidectomy connected with significant decreases in PTH (79). However, we cannot exclude the effect of habitual alcohol consumption on VitD levels in this study, even though regression models did not identify alcohol consumption as a significant predictor of VitD levels.

Probably the most difficult is to explain the positive association of vitamin D with the level of adiposity, triglycerides, and liver enzymes indicative of fatty liver disease, and the negative association with HDL-cholesterol. Most literature data contrast with these findings and show the negative association with the level of adiposity (3), blood lipids (80, 81), and liver enzymes (82), even though there are also some conflicting findings (1–4, 83).

In line with this study findings of increased prevalence of obesity and metabolic disturbances among coal miners despite their heavy physical activity, in a recent NHANES study, only increased recreational physical activity was found to be associated with an improved metabolic profile, while occupational physical activity was not (84). In contrast, in a recent study of Chinese coal miners, the higher level of occupational physical activity was related to a decreased prevalence of metabolic syndrome among them (23).

Even though most (almost 70%) of the subjects were overweight and obese (according to BMI), only less than half of them had increased adiposity, centripetal obesity, or metabolic syndrome, which can be explained by the increased muscular mass related to their heavy physical work and the effect of physical activity on waist circumference and metabolic parameters (85–87). However, in this study, hyperglycemia was detected in more than 60%, while dyslipidemia (according to at least one lipid parameter) was detected in even more than 80% of the study subjects. In accordance with this study, in a study of Indian coal miners, serum triglycerides, total cholesterol, very-low-density lipoproteins (VLDL)-cholesterol, and LDL- cholesterol levels were found to be increased, while HDL- cholesterol levels were found to be decreased, in the coal miners compared with other workers, irrespective of the level of adiposity, which suggests that some other factors (inappropriate diet or working conditions, e.g., shift work) additionally contribute to metabolic disturbances (22).

In this study, the most prevalent type of dyslipidemia was hypertriglyceridemia. However, the levels of HDL-cholesterol were not decreased in the same magnitude (only in one-quarter of the subjects were they decreased), which can be explained by the positive effect of heavy physical activity on HDL-cholesterol, which is more sensitive to physical activity compared with LDL-cholesterol and triglycerides (87).

The positive association of serum 25 (OH) vitamin D levels with the levels of adiposity in this study can be explained by increased lipolysis and VitD release from adipose tissue depots during and after heavy physical work (60). Consistent with this, in the study of Sun et al. (64), increased 25 (OH) VitD levels after physical activity persisted for 24 h, and the strongest positive predictor of the incremental area under the curve was %FM, which is in agreement with the here presented findings. In this study, there were also positive correlations of VitD levels with limb muscle mass (although on the borderline statistical significance), which also confirms that muscular depots contribute to VitD status, although to a lesser extent (60–62).

In adipose tissue, most of the stored vitamin D is in its non-hydroxylated form (60). After the non-hydroxylated form of VitD is released from fat and muscular depots, it binds to a VitD binding protein, (DBP) and enters the liver where it is activated (25–hydroxylated) and, without delay, is released again into circulation, to be stored again in fat and muscular depots (and other target organs), with a half-life of ~2–3 weeks, which ensures that the circulating 25 (OH) VitD levels are quite stable and not significantly influenced by transient dietary intake and sun exposure changes (60). This is in accordance with the findings of this study that the previous day VitD intake did not correlate with the measured 25 (OH) VitD levels.

The negative correlation of vitamin D status with age in this study is in agreement with previously published data since older people produce less cutaneous VitD3 (46), and we have used age as a controlling factor to examine the association of VitD with other parameters. Since in this study both VitD status and different types of dyslipidemia had a positive association with the level of adiposity (but not centripetal obesity), we used adiposity parameters (FM and FM%) as additional controlling factors. In the adjusted analyses, the associations with TG, HDL, lipid ratios, ALT, and creatinine remained significant, as well with leg FM%. Only after additionally correcting for leg FM% was the significance of these associations with biochemical parameters lost, indicating that leg FM% was an additional factor affecting the association of VitD and biochemical parameters. Similarly, in the regression analysis, the only significant predictors of VitD status were age, leg FM%, and triglyceride levels.

The association with leg FM% can be explained by the fact that leg (femoral) fat depots represent an important resource of lipolysis during coal mining, even though the upper body subcutaneous fat generally contributes more to lipolysis in men during rest and exercise (88, 89). However, there are some conflicting data, indicating that in younger overweight men (in contrast to older overweight men) femoral fat depots respond more to exercise by lipolysis, compared with abdominal fat depots, which was related to the initial size of the depot (89, 90). In addition, even though leg fat is considered to have a more protective metabolic function and to be associated with a more beneficial metabolic profile (88), the findings from this study indicate the opposite. In this study, different types of dyslipidemia had a positive association with the level of adiposity, but, surprisingly, more with indicators of peripheral (gluteo-femoral) rather than centripetal (truncal) adiposity (e.g., lipid levels more correlated with hip circumference and leg FM% than with waist circumference and trunk FM%).

Unfortunately, there are not enough data on the difference between distinct subcutaneous and visceral fat depots in the storage and release capacity for VitD, particularly on the difference between distinct subcutaneous depots (60, 91). In one study (91), the concentrations of the non-hydroxylated form of VitD in visceral fat depots were moderately higher compared with abdominal subcutaneous fat depots, in both obese and non-obese women (with no difference between them). Nevertheless, considering a significantly larger amount of subcutaneous fat depots (4–6-fold), these depots contribute the most to circulating VitD levels (60).

On the other hand, the association of vitD with adipose tissue is bidirectional, and vitD can influence fat accumulation and re-distribution: in one study (92), supplementation with VitD decreased truncal subcutaneous fat without changing total fat mass, and this effect can be attributed to the direct effects of VitD on adipose tissue (probably through adipose tissue VitD receptor actions, or through its effects on calcium metabolism) and lower PTH levels (93–96).

Since high 25 (OH) vitD3 levels have a reverse relationship with PTH levels (78), we suppose that in the population of coal miners (with high VitD levels), the levels of PTH were lower than in the general Serbian male population. PTH is known to simulate truncal fat distribution, thus centripetal obesity and related metabolic disturbances (i.e., metabolic syndrome) are often observed in primary hyperparathyroidism (79, 97–99). Therefore, lowering the levels of PTH can lead to a more favorable peripheral fat distribution and metabolic profile (79, 98, 99). Increased/decreased PTH secretion can be a possible explanation for commonly observed negative relationships of serum VitD with centripetal obesity and metabolic syndrome (28) and the in this study found positive association with peripheral fat accumulation (hip circumference and leg FM%). Unfortunately, we did not measure the levels of PTH in this study, as it was not originally planned in the study design to measure PTH, and even though (after measuring VitD) we had some leftovers of the serum samples of coal miners, with an adequate quantity for PTH measurements, the low stability of PTH in the serum samples during long-term storage (100) did not allow us to perform reliable measurements. Nevertheless, in this study, three-quarters of the coal miners had a centripetal body fat distribution (according to WHR), despite the very high levels of VitD (and probably low levels of PTH), and there was no association of VitD with WHR or waist circumference, which indicates that the role of VitD and PTH on body fat distribution was insignificant in the coal miners. This is in accordance with data on the imperceptible effects of VitD on obesity treatment (94, 101) and with data that show that in subjects with high levels of VitD, there is no effect of additional supplementation with VitD on body fat re-distribution (96, 101).

Particularly interesting is the finding of a positive association of serum 25 (OH) VitD levels with serum triglycerides levels. In general, most of the published data (but not all) indicate a negative association of VitD with triglyceride levels, and a positive association with HDL-cholesterol levels (80, 81). Triglycerides and HDL-cholesterol generally have an inverse association (102, 103), which we confirmed in this study (Spearman's coefficient of correlation = −0.615, *p* < 0.001), and the linear regression analysis identified only triglycerides as significant predictors of VitD levels. Interestingly, and in line with this study findings, some studies show that VitD supplementation increased the levels of triglycerides (104–106), even though both unchanged (107–109) and decreased (110) levels were found in other studies. Therefore, we hypothesize that the observed positive relationship of VitD with serum triglycerides reflects the effect of positive association with lipolysis after an enduring physical work (89). The increased lipolysis during prolonged physical work not only increases the release of VitD from adipose and muscular depots but also increases the supply of free fatty acids (FFAs), released from fat tissue, to the liver. In the liver, accumulated FFAs stimulate the production and secretion of VLDL, the main transporters of serum triglycerides, which are consequently cleared, mostly by muscle and adipose tissue (89, 111).

In general, under fasting and resting conditions, circulating triglycerides are in direct proportion with the rates of release and oxidation of FFAs (111). After physical activity, lipolysis is still increased, and this effect can last up to 24 to 42 h (89, 112, 113). The increased FFAs' release after physical activity increase the FFAs' supply to the liver. The increased supply of FFAs to the liver increases triglycerides secretion from the liver (in the form of VLDL) (111), but one could expect that triglycerides will be rapidly cleared from circulation by lipoprotein lipase (LPL), whose activity in a muscle is concomitantly increased during recovery after a physical activity (87), which would lead to a drop in triglycerides levels. However, quite the opposite, increases in triglyceride levels have also been described several hours after physical activity, indicating that their increased production can prevail over their increased clearance (87, 114). In accordance, the increased FFAs supply to muscle and fat tissue after physical activity can interfere with triglyceride clearance by LPL (111). Alcohol consumption additionally decreases LPL activity (115). Finally, studies show that total energy replacement after physical activity (what coal miners do after their work) diminishes/completely abolishes the beneficial effects of the physical activity on the next-day fasting and postprandial triglyceride and HDL-cholesterol levels (87, 114, 116, 117).

Nevertheless, these hypotheses need to be confirmed by further studies, and there can also be other mechanisms of the found associations of VitD levels with cardiometabolic parameters.

In accordance with the present study findings of high vitD values associated with worse metabolic profile among coal miners, some studies show a U-shaped association of VitD levels with cardiovascular mortality and risk, which requires further investigation (46).

Although not a primary aim of this study, the positive correlation of VitD with creatinine can be explained by the confounding effect of increased physical activity and release from muscular depots, since after a heavy physical activity the levels of creatinine also increase (118, 119), apart from the levels of VitD. Similarly, after heavy physical activity, the levels of ALT, AST, and GGT increase and can stay increased even after 24 h (118). The positive correlation with erythrocyte number could be explained by the direct effects of VitD on erythropoiesis and/or erythrocytes survival (120, 121), but can also be another confounding effect of physical activity (connected with increased erythrocyte release, due to splenic contraction, and hemoconcentration) (122).

This study has several limitations. The main limitation of this study is that only the late summer VitD levels are measured, without additional measurements of the winter/early spring VitD levels [which are expected to be much lower (19, 20)]. An additional limitation is the non-existence of a direct control group of non-coal miners from the same area, matched by sex, age, and anthropometric variables. However, we compared the data with recent literature findings for the Serbian general male population (urban-living and not working in physically demanding jobs but with similar age and anthropometric features), even though the method for VitD measurement (chemiluminescent immunoassay, CLIA) and the month and year of study sampling (July 2014) were not the same (28). Nevertheless, we had some serum samples from a small group of urban-living general population from the same latitude, from the beginning of September 2016 (the same as in the present study) and analyzed by HPLC at the same time with the samples from the coal miners, but that group was very small and mostly made of women (22 women and two men), and the data only served for HPLC method verification. The mean values in this group were 55.1 ± 30.9 nmol/L, again almost three times lower than in the coal miners and similar to data in reference (28). Another limitation is that the sample of the miners was not randomly taken (as it was a part of systematic medical examinations of all employed coal miners from one coal mine/shaft in the Resavica basin) and was only from one region for coal mining in Serbia, but we think that also in other regions situation will be quite similar (since they are very near geographically), and the most of Serbian coal miners work in the sampled region (76.4%) (27). Furthermore, we performed HPLC, but liquid chromatography-tandem mass spectrometry (LC-MS/MS) is the gold standard for VitD measurement (46, 78). We did not measure PTH levels, nor the free (i.e., bioavailable, not bound with DBP) VitD form, the active form of VitD (1,25 (OH)_2_ VitD), other VitD metabolites or epimers (e.g., C-3 epimers), which could modify or more explain these results (63, 78). In addition, some data show that VACUETTE® tubes with a serum clot activator can interfere with immunoassays for 25 (OH) VitD measurements (123, 124), but there are no such data on the HPLC method with Vacutest Kima® tubes with a serum clot activator (125), and even though a bit higher values were observed in immunoassays with VACUETTE® tubes with a serum clot activator, they correlated well with the ones measured by LC-MS/MS (123, 124). Moreover, TANITA BIA segmental analysis is not that accurate and precise in assessing regional body composition as other methods (e.g., dual-energy X-ray absorptiometry, DXA; computerized tomography, CT; magnetic resonance imaging, MRI), particularly in some populations (126). However, the mentioned techniques are costly and cannot be easily applied in field settings, and there is still a fairly good estimation with an eight-contact electrode BIA system, as confirmed by some validity studies (34). Finally, we did not directly measure or precisely estimate the level of individual physical activity, since it was not initially planned. Nevertheless, we controlled for physical activity in the questionnaire. All the coal miners were asked about their self-reported physical activity levels and the specific jobs they perform during coal mining. All of them were involved in hewing coal, loading coal, or (to a much lesser extent) erecting roof supports, and were placed in the heavy occupational work physical activity level (PAL) category (31). There are some publications which describe physical activities and directly measure physical activity and energy expenditures during coal mining (25, 127), and there was no huge difference between hewing coal and loading coal in energy expenditure. Nevertheless, it would be of interest to directly measure or at least precisely estimate the individual level of physical activity, and to relate it to individual VitD status in coal miners, to support the presented hypotheses.

Coal miners are a population of workers with specific nutritional needs due to their heavy physical work and unfavorable working conditions: working in shifts, working underground, and working with chronic occupational exposure to coal mine dust, which has pro-oxidative, pro-inflammatory, and pro-cancerogenic properties (128–130). Therefore, in the view of the science of precision nutrition, measuring the levels of VitD (which has the important metabolic, antioxidant, and anti-inflammatory activities) is very important in this population of workers to achieve their optimal nutrition, to adjust their nutrition (and possible supplementation) and meet their specific nutritional needs (131).

Considering the unexpected findings of this study, which are not easy to explain, and the above-mentioned study limitations, more studies are warranted to confirm the results and further explore the topic of VitD status and cardiometabolic health among coal miners.

## Conclusion

In this study, we have found surprisingly high levels of late summer serum 25 (OH) VitD in a population of Serbian coal miners, much higher compared with the same season VitD data from an urban-living Serbian general male population with similar anthropometric characteristics, and previously published data for coal miners from other countries. Furthermore, we have found unexpected positive correlations of serum 25 (OH) VitD levels with anthropometric and biochemical parameters indicative of obesity, metabolic syndrome, and fatty liver disease. The linear regression analysis identified that the most significant positive predictors of VitD levels were age, leg FM%, and triglyceride levels. Such unexpected findings require more exploration. Data on VitD levels in the population of coal miners are scarce, as well as data on their cardiometabolic health. More research is needed on the VitD status of coal miners (particularly in the winter period) and its relationship with their cardiometabolic status.

## Data Availability Statement

The raw data supporting the conclusions of this article will be made available by the authors, without undue reservation.

## Ethics Statement

The studies involving human participants were reviewed and approved by Institute of Occupational Health Niš Ethics Board. The patients/participants provided their written informed consent to participate in this study.

## Author Contributions

IS wrote the manuscript and performed anthropometric measurements, nutritional interviews, software exploration of nutritional data, biochemical analyses, and all the statistical analyses. JJ organized and performed preventive medical examinations, clinical assessments of coal miners, and biochemical analyses. MZec and ZP performed VitD measurements by HPLC. JD-M performed biochemical analyses. MZek and JM were involved in the dietary assessment. MGu and MGl led the project under which the study was conducted. All authors contributed to the writing of the manuscript.

## Conflict of Interest

The authors declare that the research was conducted in the absence of any commercial or financial relationships that could be construed as a potential conflict of interest.

## Publisher's Note

All claims expressed in this article are solely those of the authors and do not necessarily represent those of their affiliated organizations, or those of the publisher, the editors and the reviewers. Any product that may be evaluated in this article, or claim that may be made by its manufacturer, is not guaranteed or endorsed by the publisher.
