# Supplementary material for: Vitamin D Status and Its Correlation With Anthropometric and Biochemical Indicators of Cardiometabolic Risk in Serbian Underground Coal Miners in 2016
Source: Front Nutr. 2021 Aug 19;8:689214. doi: 10.3389/fnut.2021.689214 (PMC8417231; doi:10.3389/fnut.2021.689214)
Supplement: Supplementary file 1 [file Data_Sheet_1.DOCX]

Supplementary Material

# Supplementary Figures and Tables

## Supplementary Figures


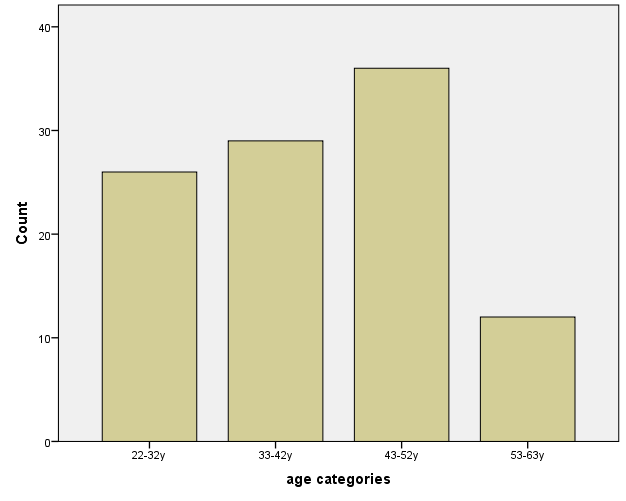


**Supplementary Figure 1.** Distribution of coal miners (N=103) across 10-years age categories


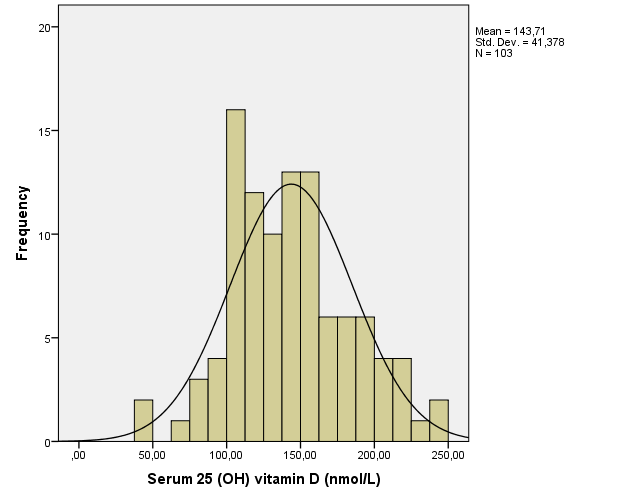


**Supplementary Figure 2.** Histogram of serum 25 (OH) vitamin D values in the studied group of coal miners (N=103)

## Supplementary Tables

**Supplementary Table S1. Correlation of hematological and biochemical data with coal miners’ anthropometric characteristics (*N* =103, males)**

|  | **Age** | **Body weight** | **BMI** | **FM** | **FM%** | **FFM** | **Muscle mass** | **Skeletal mass** | **Arms FM%** | **Trunk FM%** | **Legs FM%** | **Arms muscle mass** | **Trunk muscle mass** | **Legs muscle mass** | **VFL** | **Waist circumference** | **Hip circumference** | **WHR** |
| --- | --- | --- | --- | --- | --- | --- | --- | --- | --- | --- | --- | --- | --- | --- | --- | --- | --- | --- |
|  | *r_s_* | | | | | | | | | | | | | | | | | |
| **ESR** | **0.238^*^** | -0.123 | -0.003 | -0.034 | 0.012 | -0.172 | -0.175 | -0.180 | 0.026 | 0.029 | -0.016 | -0.190 | -0.168 | -0.150 | 0.099 | -0.029 | -0.119 | 0.101 |
| **WBC** | -0.030 | -0.059 | -0.004 | -0.004 | 0.047 | -0.106 | -0.105 | -0.114 | 0.037 | 0.052 | 0.052 | -0.099 | -0.106 | -0.108 | 0.038 | -0.016 | -0.097 | 0.079 |
| **RBC** | -0.156 | 0.022 | -0.026 | 0.116 | 0.137 | -0.066 | -0.062 | -0.060 | 0.115 | 0.116 | 0.174 | -0.055 | -0.081 | -0.051 | 0.021 | 0.033 | 0.009 | 0.008 |
| **HGB** | -0.089 | -0.003 | 0.004 | 0.082 | 0.125 | -0.093 | -0.091 | -0.083 | 0.079 | 0.106 | 0.176 | -0.068 | -0.122 | -0.070 | 0.038 | 0.012 | -0.017 | -0.004 |
| **HCT** | 0.063 | -0.053 | -0.048 | 0.081 | 0.152 | -0.173 | -0.169 | -0.168 | 0.091 | 0.144 | 0.179 | -0.169 | -0.198^*^ | -0.160 | 0.098 | -0.005 | -0.095 | 0.025 |
| **MCV** | **0.277^**^** | -0.098 | -0.015 | -0.095 | -0.052 | -0.098 | -0.101 | -0.103 | -0.074 | -0.028 | -0.094 | -0.123 | -0.103 | -0.104 | 0.069 | -0.074 | -0.100 | -0.022 |
| **MCH** | 0.108 | -0.041 | 0.039 | -0.078 | -0.056 | -0.025 | -0.028 | -0.029 | -0.073 | -0.047 | -0.061 | -0.014 | -0.035 | -0.029 | 0.028 | -0.053 | -0.021 | -0.059 |
| **MCHC** | **-0.223^*^** | 0.124 | 0.137 | 0.055 | 0.007 | 0.139 | 0.137 | 0.149 | 0.032 | <0.001 | 0.031 | 0.179 | 0.128 | 0.165 | -0.048 | 0.058 | 0.121 | -0.013 |
| **PLT** | -0.144 | **-0.247^*^** | **-0.266^**^** | **-0.277^**^** | **-0.250^*^** | -0.176 | -0.175 | -0.178 | **-0.275^**^** | **-0.250^*^** | **-0.221^*^** | -0.168 | -0.140 | **-0.220^*^** | **-0.278^**^** | **-0.201^*^** | **-0.290^**^** | 0.016 |
| **Glucose** | 0.179 | **0.254^**^** | **0.268^**^** | **0.248^*^** | **0.233^*^** | 0.174 | 0.172 | 0.167 | **0.226^*^** | **0.217^*^** | **0.229^*^** | 0.162 | 0.164 | **0.202^*^** | **0.262^**^** | **0.236^*^** | 0.132 | **0.235^*^** |
| **Triglycerides** | 0.120 | 0.167 | **0.209^*^** | **0.240^*^** | **0.265^**^** | 0.101 | 0.102 | 0.099 | **0.227^*^** | **0.237^*^** | **0.323^**^** | 0.111 | 0.085 | 0.075 | **0.263^**^** | 0.175 | 0.192 | 0.083 |
| **Total cholesterol** | 0.139 | -0.019 | 0.015 | 0.083 | 0.132 | -0.085 | -0.085 | -0.082 | 0.078 | 0.073 | **0.216^*^** | -0.068 | -0.083 | -0.104 | 0.089 | -0.004 | 0.005 | -0.018 |
| **LDL-cholesterol** | 0.153 | 0.046 | 0.038 | 0.131 | 0.166 | -0.028 | -0.029 | -0.031 | 0.109 | 0.108 | **0.245^*^** | -0.006 | -0.020 | -0.061 | 0.140 | 0.035 | 0.062 | 0.009 |
| **HDL-cholesterol** | -0.007 | **-0.303^**^** | **-0.260^**^** | **-0.247^*^** | **-0.214^*^** | **-0.283^**^** | **-0.285^**^** | **-0.273^**^** | -0.187 | -0.183 | **-0.313^**^** | **-0.304^**^** | **-0.273^**^** | **-0.215^*^** | **-0.263^**^** | **-0.257^**^** | **-0.238^*^** | -0.187 |
| **TC/HDL** | 0.115 | **0.211^*^** | 0.193 | **0.246^*^** | **0.256^**^** | 0.155 | 0.156 | 0.148 | **0.203^*^** | **0.196^*^** | **0.371^**^** | 0.177 | 0.146 | 0.087 | **0.266^**^** | 0.182 | 0.190 | 0.111 |
| **LDL/HDL** | 0.120 | 0.193 | 0.163 | **0.241^*^** | **0.254^**^** | 0.125 | 0.126 | 0.117 | 0.197^*^ | 0.189 | **0.363^**^** | 0.153 | 0.116 | 0.058 | **0.254^**^** | 0.163 | 0.165 | 0.118 |
| **TG/HDL** | 0.097 | **0.234^*^** | **0.251^*^** | **0.267^**^** | **0.273^**^** | 0.178 | 0.180 | 0.174 | **0.235^*^** | **0.239^*^** | **0.356^**^** | **0.196^*^** | 0.164 | 0.130 | **0.293^**^** | **0.219^*^** | **0.227^*^** | 0.125 |
| **AST** | -0.131 | 0.124 | 0.141 | 0.093 | 0.045 | 0.116 | 0.117 | 0.127 | 0.062 | 0.033 | 0.067 | 0.136 | 0.088 | 0.182 | -0.008 | 0.136 | 0.084 | 0.129 |
| **ALT** | -0.099 | **0.416^**^** | **0.408^**^** | **0.389^**^** | **0.343^**^** | **0.354^**^** | **0.355^**^** | **0.362^**^** | **0.315^**^** | **0.304^**^** | **0.438^**^** | **0.379^**^** | **0.324^**^** | **0.374^**^** | **0.282^**^** | **0.430^**^** | **0.361^**^** | **0.356^**^** |
| **ALT/AST** | -0.033 | **0.446^**^** | **0.412^**^** | **0.428^**^** | **0.403^**^** | **0.375^**^** | **0.376^**^** | **0.374^**^** | **0.359^**^** | **0.364^**^** | **0.499^**^** | **0.389^**^** | **0.358^**^** | **0.355^**^** | **0.363^**^** | **0.454^**^** | **0.407^**^** | **0.354^**^** |
| **GGT** | 0.137 | **0.281^**^** | **0.318^**^** | **0.331^**^** | **0.355^**^** | **0.201^*^** | **0.201^*^** | **0.204^*^** | **0.292^**^** | **0.314^**^** | **0.414^**^** | **0.233^*^** | **0.176** | **0.199^*^** | **0.331^**^** | **0.373^**^** | **0.252^*^** | **0.414^**^** |
| **ALP** | **-0.256^**^** | 0.036 | 0.008 | 0.022 | -0.008 | 0.048 | 0.048 | 0.047 | 0.004 | 0.015 | -0.014 | 0.050 | 0.017 | 0.081 | -0.042 | 0.035 | 0.032 | 0.016 |
| **Total bilirubin** | 0.040 | 0.060 | 0.042 | 0.091 | 0.058 | 0.022 | 0.024 | 0.020 | 0.070 | 0.049 | 0.061 | <0.001 | 0.032 | 0.026 | 0.109 | 0.027 | 0.064 | -0.058 |
| **Urea** | 0.168 | 0.040 | 0.013 | 0.021 | 0.015 | 0.055 | 0.053 | 0.052 | 0.029 | 0.012 | 0.033 | 0.019 | 0.067 | <0.001 | 0.031 | 0.026 | 0.012 | 0.064 |
| **Creatinine** | -0.149 | 0.170 | 0.142 | 0.059 | 0.014 | **0.238^*^** | **0.241^*^** | **0.243^*^** | 0.048 | -0.027 | 0.111 | **0.235^*^** | **0.252^*^** | **0.209^*^** | -0.056 | 0.088 | 0.184 | -0.031 |

* *p* < 0.05; correlation is significant at the 0.05 level (2-tailed).

** *p* < 0.01; correlation is significant at the 0.01 level (2-tailed).

Note: BMI = Body Mass Index; FM = Fat mass; FM% = Percentage of fat mass; FFM = Fat-free mass; VFL = visceral fat level; WHR = waist to hip ratio; BP = Blood Pressure; LDL = Low Density Lipoprotein; ESR = erythrocyte sedimentation rate; WBC = white blood cell count; RBC = red blood cell count; HGB = hemoglobin; HCT = hematocrit; MCV= mean volume; MCH = mean corpuscular hemoglobin; MCHC = mean corpuscular hemoglobin concentration; PLT = platelet count; HDL = High Density Lipoprotein; TC/HDL = Total cholesterol / HDL-cholesterol ratio; LDL/HDL = LDL-cholesterol / HDL-cholesterol ratio; TG/HDL = Triglycerides / HDL-cholesterol ratio; AST = aspartate aminotransferase; ALT = alanine aminotransferase; ALP = alkaline phosphatase; GGT = gamma glutamyl transferase; *r_s_* = Spearman’s rank correlation coefficients; *p* = statistical significance of correlations.
